# An update on methods for detection of prognostic and predictive biomarkers in melanoma

**DOI:** 10.3389/fcell.2023.1290696

**Published:** 2023-10-13

**Authors:** Oluwaseyi Adeuyan, Emily R. Gordon, Divya Kenchappa, Yadriel Bracero, Ajay Singh, Gerardo Espinoza, Larisa J. Geskin, Yvonne M. Saenger

**Affiliations:** ^1^ Columbia University Vagelos College of Physicians and Surgeons, New York, NY, United States; ^2^ Albert Einstein College of Medicine, Bronx, NY, United States; ^3^ Department of Dermatology, Columbia University Irving Medical Center, New York, NY, United States

**Keywords:** immunotherapy, checkpoint inhibition, predictive biomarker, prognostic biomarker, melanoma

## Abstract

The approval of immunotherapy for stage II-IV melanoma has underscored the need for improved immune-based predictive and prognostic biomarkers. For resectable stage II-III patients, adjuvant immunotherapy has proven clinical benefit, yet many patients experience significant adverse events and may not require therapy. In the metastatic setting, single agent immunotherapy cures many patients but, in some cases, more intensive combination therapies against specific molecular targets are required. Therefore, the establishment of additional biomarkers to determine a patient’s disease outcome (i.e., prognostic) or response to treatment (i.e., predictive) is of utmost importance. Multiple methods ranging from gene expression profiling of bulk tissue, to spatial transcriptomics of single cells and artificial intelligence-based image analysis have been utilized to better characterize the immune microenvironment in melanoma to provide novel predictive and prognostic biomarkers. In this review, we will highlight the different techniques currently under investigation for the detection of prognostic and predictive immune biomarkers in melanoma.

## Introduction

The yearly incidence of skin cancer in the United States is greater than all other types of cancer combined (O’Neill and Scoggins, 2019; Dzwierzynski, 2021). Among skin cancers, melanoma is the most aggressive. An individual’s lifetime risk of developing melanoma has gone from 1 in 500 in 1935 to 1 in 50 in 2023, partially due to increased awareness and early detection of disease (Volkovova et al., 2012; Rastrelli et al., 2014; Dzwierzynski, 2021). Thus, the need for better prevention and treatment of this disease is increasingly critical.

The development of immunotherapy has served as a pivotal turning point in the treatment of many cancers and melanoma in particular (Hu-Lieskovan et al., 2020). Specifically, the discovery of antibodies directed to immune checkpoint molecules such as programmed cell death protein 1 (PD-1) and cytotoxic T-lymphocyte-associated-protein 4 (CTLA-4), and, more recently, lymphocyte activation gene 3 (LAG-3), have drastically prolonged survival in melanoma (Uhara, 2019; Huuhtanen et al., 2023). Ipilimumab, an anti-CTLA-4 antibody, was FDA approved for the treatment of unresectable or metastatic melanoma in 2011 based on phase 3 data showing superiority to chemotherapy and treatment with a well-studied vaccine against glycoprotein 100 (gp100) and was subsequently found to also show promise in the adjuvant setting (Hodi et al., 2010; Robert et al., 2011; Sarnaik et al., 2011; Sanlorenzo et al., 2014). Anti-PD-1 antibodies, however, have become the gold standard treatment for melanoma based on studies comparing anti-PD-1 to anti-CTLA-4 and to chemotherapy (Topalian et al., 2012). A recent study found that pembrolizumab, an anti-PD-1 antibody, may be used as adjuvant therapy in stage IIB and IIC melanoma, in addition to advanced melanoma and resected stage III disease (Luke et al., 2022). There is now very strong data to support neoadjuvant therapy for stage III melanoma, although integrating with surgical management in small volume disease may not always be straightforward and this is currently an area of investigation (Patel et al., 2023). Combination anti-CTLA-4 and anti-PD-1 is more effective than either alone but at the cost of significant toxicity (Wolchok et al., 2013). Most recently, the combination of anti-PD-1 and anti-LAG-3 gained FDA approval, providing a second immunotherapy option, although prolonged overall survival (OS) with this regimen is not yet proven (Huuhtanen et al., 2023). Emerging immune checkpoint molecules [e.g., adenosine A2A receptor (A2AR), T cell immunoglobulin and mucin domain 3 (TIM3), V-domain Ig suppressor of T cell activation (VISTA)] are being explored in clinical trials with already approved ICIs, offering potentially additional treatment options (Hu-Lieskovan et al., 2020). Tumor-infiltrating lymphocyte (TIL) therapy has also been shown to have high efficacy rates as first-line therapy as well as in the post-PD-1 setting in phase II and phase III trials, but has not yet reached regulatory approval (Rohaan et al., 2022; Monberg et al., 2023).

The notable phase III DREAMseq trial established that immunotherapy should precede targeted therapy for patients with treatment-naive *BRAF V600*-mutant metastatic melanoma (Atkins et al., 2023). Thus, immunotherapy has become the standard of care for resected stage III melanoma, and most recently for resected stage IIB-C melanoma. Immunotherapy is FDA approved despite the fact that over 75% of patients are cured by surgery alone. Therefore, the need for biomarkers focuses on two areas: selecting patients with advanced disease for combination immunotherapy and selecting patients with early stage disease for single-agent adjuvant immunotherapy.

Although dramatic success has come from the advent of immune checkpoint inhibitors (ICI), these therapies have also been associated with multiple adverse events including elevated liver enzymes, rash, pruritus and fatigue, among many others. Grade 3 or 4 adverse events have also been commonly reported, oftentimes in over 50% of melanoma patients treated in clinical trials with ICI (Wolchok et al., 2010; Robert et al., 2011; Wolchok et al., 2013). Given these significant clinical findings, as well as the expense and inconvenience incurred by treatment, the establishment of biomarkers will be crucial in helping clinicians better weigh the potential response to immunotherapy against the potential risks and adverse effects.

Biomarkers are measurable biological indicators that can be subdivided into two main groups: prognostic and predictive (Nalejska et al., 2014; Rizk et al., 2019). While prognostic biomarkers indicate a patient’s disease outcome regardless of treatment, predictive biomarkers are used to estimate how likely a patient will respond to a given therapy. Thus, prognostic biomarkers may identify high-risk patients that may benefit from multimodal, more aggressive therapy while predictive biomarkers may indicate a patient’s specific treatment outcome. To discover these biomarkers, multiple techniques have been utilized including gene expression profiling of bulk tissue and spatial transcriptomics of single cells, among others. This review summarizes these different techniques from the current standard to newer technological areas of innovation such as multiplexing and artificial intelligence (AI)-based image analysis for the development of prognostic and predictive biomarkers in melanoma and highlights directions for future biomarker development.

## Current gold standard

Traditionally, the major clinical and histological prognostic biomarkers of melanoma have included primary tumor (i.e., Breslow) thickness, ulceration, mitotic rate, anatomic site (i.e., acral, cutaneous, mucosal or uveal) and sentinel lymph node (SLN) involvement (Balch et al., 2001; Abbas et al., 2014). These factors have been largely captured in the Tumor, Node and Metastasis (TNM) system by the American Joint Committee on Cancer (AJCC). In the current (eighth) edition of the TNM system, Breslow thickness and ulceration, which comprise the T category, continue to be correlated with survival with thicker and ulcerated tumors more associated with poorer prognosis (Keung and Gershenwald, 2018; In ’t Hout et al., 2012). However, mitotic rate, which remains a strong indicator of prognosis in melanomas of varying thickness, is no longer incorporated into the AJCC melanoma staging system.

Regarding the N category, patients with “clinically occult” nodal metastasis (i.e., patients with regional node metastasis at SLN biopsy but without accompanying clinical or radiographic evidence) have been shown to have better survival than patients with clinically or radiographically evident disease. Thus, SLN biopsies are often performed in these patients as nodal status is an important independent predictor of prognosis (Cascinelli et al., 2000; van Akkooi et al., 2008; Balch et al., 2010; Gershenwald et al., 2017; Keung and Gershenwald, 2018). A positive SLN biopsy historically warranted complete lymph node dissection (CLND). However, two multicenter randomized controlled trials found that there was no significant difference in OS between immediate CLND versus nodal observation in these patients, demonstrating new prognostic implications and roles for adjuvant systemic therapies. Non-nodal regional (e.g., microsatellite, satellite or in-transit) metastases also serve as criteria for the N category and have been associated with worse prognosis (Rao et al., 2002; Van Es et al., 2008; Wilmott et al., 2012; Read et al., 2015). For the M category of the TNM system, which refers to sites of distant metastases, patients with non-visceral (e.g., subcutaneous, cutaneous, nodal) distant metastasis have a modestly better survival than those with distant metastases to other sites (Barth et al., 1995; Keung and Gershenwald, 2018).

Although prognostic biomarkers have been well-established for melanoma, predictive biomarkers are not yet routinely used for ICI in melanoma due to limited clinical utility or low sensitivity and specificity (Huang et al., 2022). However, multiple techniques have been utilized for the discovery of promising candidates which will be discussed.

## Melanoma genetics and circulating tumor DNA

Recent advancements in genetic research have provided insight into the molecular landscape of melanoma, revealing potential genetic signatures associated with prognosis and therapeutic response. Notably, a genome-wide sequencing program of thousands of melanomas worldwide revealed that mutations in *BRAF* are most common, with reported frequencies of over 60% (Akbani 2015; Tímár and Ladányi, 2022). Specifically, a substitution of valine with glutamate at residue 600 (*V600E*) accounts for over 90% of mutations within that locus (Dahl and Guldberg, 2007). The prognostic significance of *BRAF* mutations has been widely controversial as some studies have found that *BRAF*-mutated melanomas may be associated with worse survival and higher risk of recurrence but other studies have shown no survival difference compared to *BRAF* wild-type (Meckbach et al., 2014; Adler et al., 2017; Ny et al., 2020; Naimy et al., 2023). Following *BRAF*, *NRAS*, and *NF1* comprise the next most common mutations identified in melanoma. Patients with *NRAS*- and *NF1*- mutated melanomas tend to have a worse prognosis (Cirenajwis et al., 2017; Podlipnik et al., 2021; Randic et al., 2021). Mutations in *BRAF*, *NRAS*, and *NF1* represent driver mutations that lead to aberrant activation of the MAPK pathway, which is important for cell proliferation and survival (Burotto et al., 2014).

The identification of mutations guide therapy for melanoma. BRAF inhibitors (BRAFi) such as dabrafenib and trametinib have been shown to improve progression-free survival (PFS) and OS in patients with these mutations (Long et al., 2017a; Long et al., 2017b; Yang et al., 2023). However, these clinical benefits of BRAFi are often short-lived given increasing drug resistance. Activating mutations in *NRAS* also confer resistance to *BRAF*-targeted therapy (Nikolaou et al., 2012; Hawryluk and Tsao, 2014). Additionally, while patients with melanomas harboring a different mutation, *BRAF V600K*, respond to BRAFi, they have been shown to have shorter PFS compared to *BRAF V600E* mutant melanomas with this therapy but exhibit superior clinical response to ICI (Akbani 2015; Pires Da Silva et al., 2019; Yang et al., 2023). Additional genetic markers that indicate favorable ICI response include high tumor mutational burden (TMB), increased expression of inflammatory mediators, *BRAF* wild type and *BRCA2* mutants (Ho et al., 2013; Hugo et al., 2016; Goodman et al., 2017; Yang et al., 2023). Nonetheless, the key DREAMseq study found that initial treatment with combination ICI therapy is significantly better than initial treatment with targeted therapy against *BRAF* and *MEK*, even in patients with *BRAF*-mutated melanomas (Atkins et al., 2023).

Circulating tumor DNA (ctDNA) has also emerged as a promising prognostic and predictive blood-based biomarker to monitor disease status in advanced melanoma patients (Calapre et al., 2017). Melanoma, like many other solid tumors, releases DNA that may be isolated from peripheral blood which can then be analyzed using sensitive techniques such as next-generation sequencing (NGS) to recapitulate intratumoral heterogeneity, evaluate genomic evolution in response to treatment and reveal potential resistance mechanisms (Sacco et al., 2020). Prognostically, levels of ctDNA have been found to significantly correlate with clinically-relevant, serological markers of tumor burden such as S100 calcium-binding protein B (S100B), melanoma inhibitory activity (MIA) and lactate dehydrogenase (LDH) (Sanmamed et al., 2015; Calapre et al., 2017). In the predictive setting, multiple studies have shown that plasma ctDNA levels prior to the initiation of BRAFi therapy correlated with treatment response. Specifically, the BREAK trials revealed that high baseline ctDNA levels were reliably and significantly associated with lower PFS and overall response rate (ORR) to targeted therapy with dabrafenib (Ascierto et al., 2013; Santiag et al., 2016). A smaller number of studies have assessed the predictive value of ctDNA in patients treated with ICI. For example, a study in 2017 found that ctDNA levels at baseline and early during treatment with anti-PD-1 antibodies in metastatic melanoma patients accurately predicted tumor response, OS and PFS (Lee et al., 2017). More recently, another study showed that ctDNA levels may also inform treatment response to adjuvant ICI following curative resection (Tan et al., 2019; Tivey et al., 2022). Currently, ctDNA is being explored in clinical trials as a biomarker for melanoma recurrence and treatment response.

## Genomic profiling

Immune surveillance has been shown to have potential value in prognostication for many solid cancers (Fridman et al., 2010; Bindea et al., 2011). Thus, the immunoscore, a scoring system that quantitatively classifies TIL density both at the tumor center and invasive margin, was proposed as a biomarker for cancer progression. In primary melanoma, the presence of a very high number of TILs confers a more favorable prognosis (Clemente et al., 1996; Azimi et al., 2012). However, the universal clinical application of TILs has been limited due to observer variability. Additionally, the majority of early stage melanoma patients have “non-brisk” TILs, an intermediate TIL group that does not provide much prognostic information (Busam et al., 2001; Azimi et al., 2012; Sivendran et al., 2014). Development of biomarkers beyond TILs in the clinical setting has also been challenging given the requirement of formalin-fixed and paraffin-embedded (FFPE) samples for melanoma diagnosis, which compromises RNA for transcriptomic analysis (Bogunovic et al., 2009).

To address this need, Nanostring transcriptomic technology, which analyzes the expression of multiple transcripts under varying pathological or physiological states, has been used to profile a group of 446 immune-associated candidate genes in primary melanoma. A study in 2014 found that 53 out of the 446 screened genes predicted non-progression, disease-specific survival (DSS) and prolonged recurrence-free survival (RFS) in two independent cohorts of patients with resectable stage II-III melanoma (Sivendran et al., 2014). This 53-immune gene signature panel, called the melanoma immune profile (MIP), was validated in a third independent cohort of stage II-III melanoma patients, further stratifying this patient population into low- and high-risk groups for enrollment in clinical trials and/or exposure to potentially toxic ICI (Gartrell et al., 2019). The MIP differs from other genomic signatures such as the Castle Biosciences signature, the latter of which evaluated a 31-gene expression profile (GEP) test in patients with stage I-II disease where risk is generally lower. The Castle Biosciences test is based on the mesenchymal to epithelial transition, hypothesized to play a role in melanoma genesis (Zager et al., 2018). Recently, a 2023 study has prioritized the co-extraction of quality DNA and RNA from FFPE melanoma sections for large scale multi-omic analysis for future clinical utility. The study described, for the first time, the optimal approach for the procurement and testing of nucleic acids for the screening of somatic mutations, miRNA and methylation that may identify new gene signatures in archival and limited tumor tissue (Orlow et al., 2023). Genomic tests are also being explored as companion biomarkers in clinical trial settings.

## Single cell and spatial based genomics

Single-cell RNA-sequencing (scRNA-seq) analysis is a valuable method for obtaining gene expression profiles of individual cells which helps to identify different cell types and pathways involved in cancer progression and resistance (Lim et al., 2020; Maynard et al., 2020). However, the isolation of individual cells during the tissue dissociation step of scRNA-seq interferes with information regarding their native spatial organization within the tissue and relation to other neighboring cells. Spatial transcriptomics complements scRNA-seq by physically localizing gene sets upregulated by specific cell types thereby preserving spatial information (Yu et al., 2018; Longo et al., 2021). To achieve this aim, studies have found that messenger RNA (mRNA) can be captured on microarrays of spatially barcoded DNA capture probes. Complementary DNA (cDNA) can then be generated from the mRNA by reverse transcription and left affixed to the arrayed oligonucleotides on the slide, maintaining the RNA molecule’s original position in the tissue section using the unique positional molecular barcodes. Sequencing libraries and computational reconstruction usually follow to model the tissue’s spatial organization (Ståhl et al., 2016; Ahmed et al., 2022a; Piwecka et al., 2023).

A recent scRNA-seq study found that PRRT3-AS1, an important long non-coding RNA (lncRNA) that has been incorporated in prognostic models for prostate cancer, hepatocellular carcinoma and glioblastoma (GBM), may be required for tumor cell migration in melanoma, suggesting that PRRT3-AS1 is not only a potential prognostic biomarker but also a potential therapeutic target (Zhang et al., 2022; Liang et al., 2018; Fan et al., 2020; Zhang et al., 2021; Y et al., 2021). Additional lncRNA-based immune classes have been associated with survival and integrated into multi-omic panels for precision immunotherapy based on melanoma samples from The Cancer Genome Atlas (TCGA) (Yu et al., 2020). Aside from lncRNAs, studies have assessed the tumor ecosystem in primary melanoma to indicate prognosis. One study found that the composition of recurrent cellular neighborhoods (RCNs) involving stromal, tumor and immune cells significantly differs with disease stage. According to this model, a spatially confined suppressive TME develops in melanoma which is sustained by cytokine gradients upregulating MHC-II and IDO1 expression and by PD-1/PD-L1-mediated cell interactions (Nirmal et al., 2022).

Other cells in the TME analyzed using scRNA-seq include T cells and tumor-derived exosomes (TEXs). Studies have shown that the presence of *CXCL13*+ CD4^+^ T cells and *CXCL13* expression broadly correlates with OS in a cohort of melanoma patients, independent of immunotherapy type (Litchfield et al., 2021; Veatch et al., 2022). A TEX-related signature, termed TEXscore, using scRNA-seq was associated with shorter OS across 12 cancer types, including melanoma (Wu et al., 2021).

Predictive biomarkers have also been proposed using sc-RNAseq and spatial profiling. One study’s spatial distribution analysis found that proximity of PD-L1+ cells to tumor cells and intratumoral CD8^+^ density predicts response to ICI in the metastatic setting (Gide et al., 2020). Similar analyses have provided insight into the mechanism for resistance of melanoma cells to ICI. For example, using single-cell functional proteomics, it was discovered that certain signaling networks become activated shortly after BRAF inhibition and before the emergence of drug-resistant phenotypes (Su et al., 2017). By leveraging single-cell profiles to understand tumoral heterogeneity and putative interactions between stromal-derived factors and immune mediators within melanoma, multiple studies have called for therapeutic strategies that account for specific tumor cell composition rather than bulk tumor expression (Tirosh et al., 2016).

Moreover, sc-RNAseq has been paired with single cell T cell receptor sequencing (sc-TCRseq) to elaborate additional predictive information in melanoma, which has recently been shown to be a feasible technique in both fresh and frozen tissue (Wang et al., 2023). This paired technique allows for the simultaneous analysis of T cell clones and phenotypes within single cells, which may provide information on T cell differentiation, specificity and activation to better understand underlying disease etiology and guide future treatment strategies (Pai and Satpathy, 2021). One study found that two pretreatment characteristics in the peripheral blood—activated CD4 memory T (T_M_) cell abundance and TCR diversity—constitute promising biomarkers of ICI-induced immune-related adverse events (irAEs) in metastatic melanoma patients. Additionally, the authors identified a notable correlation between early T cell clonal expansion and the onset of severe irAEs in patients treated with combination ICI (Lozano et al., 2022). A subsequent study found that metastatic melanoma patients who responded to anti-LAG-3 and anti-PD-1 combination therapy had higher baseline TCR clonality with CD8+LAG-3+ clones that expanded and shifted to a more cytotoxic phenotype resembling NK cells (Huuhtanen et al., 2023). These studies have exemplified the versatility of sc-RNAseq across different modalities for the management of melanoma.

## Multiplexed IF

Typical approaches to immunohistochemistry (IHC) evaluation of tissue from melanoma patients have several limitations including inter-observer variability and the labeling of just a single biomarker for each tissue section. Emerging techniques, namely, multiplex IHC or immunofluorescence (mIHC/IF), have attempted to address these limitations by detecting multiple biomarkers in a single tissue section through high-throughput staining and quantitative analysis (Tan et al., 2020; Ugolini et al., 2022; Yaseen et al., 2022). This technology deters from using a cocktail of antibodies reared in separate hosts and instead relies on cycles of single antibody stains added in sequential order, which are subsequently removed in order for the next antibody to be added without cross-reaction (Nguyen et al., 2021). Studies utilizing mIHC/IF have focused largely on identification of specific cell populations in the melanoma tumor microenvironment (TME) to evaluate prognosis and assess response to melanoma immunotherapies.

TILs, a major component of the TME, have been implicated in the prevention or progression of tumor growth and invasion leading to significant interest in TILs as a potential prognostic biomarker (Oble et al., 2009; Gartrell et al., 2018; Rizk et al., 2019; Gartrell-Corrado et al., 2020). Conventional IHC methods have found that as melanocytic lesions transform from benign nevi to malignant melanomas, the absolute number of TILs rises (Hussein et al., 2006; Rizk et al., 2019). The use of mIHC has elaborated on these findings to show that the presence of TILs, particularly in the stroma, is a favorable prognostic indicator (Gartrell et al., 2018). Aside from TILs, melanoma-associated tertiary lymphoid structures (TLS) are associated with improved OS and lower risk of tumor recurrence following metastasectomy (Lynch et al., 2021; Mauldin et al., 2021).

Regarding predicting response to ICI, a multiplex chromogenic and IF study of melanoma samples showed that proximity between PD-1 and PD-L1+ cells was associated with response to anti-PD-1 therapy. Similarly, high co-localization of PD-L1 and CD8 expression was associated with increased response to targeted immunotherapy (Tumeh et al., 2014). In another study, depleting mast cells in the TME was found to improve responsiveness to anti-PD-1 therapy (Somasundaram et al., 2021).

Further, the AstroPath platform, a multistep framework for multispectral mIF, produces high quality datasets at the single cell level for biomarker development and quantitative pathology to inform precision ICI. Leveraging concepts drawn from the field of astronomy, this study was able to classify PD-1 and PD-L1 expression intensity on different cell types in the TME *in situ* on pretreatment melanoma specimens from advanced melanoma patients on ICI. In this study, higher density of early effector T cells (CD8+FoxP3+) correlated with response to anti-PD-1 therapy whereas the CD163+PD-L1- myeloid phenotype was associated with lack of response to PD-1 blockade (Berry et al., 2021).

Multiple studies have also integrated mIHC/IF with additional technologies to identify predictive biomarkers. Digital spatial profiling (DSP) with multiplex IF demonstrated that PD-L1 expression in macrophages but not tumor cells was a predictive marker for PFS, OS, and treatment response. Further, specific immune markers associated with PFS and OS, respectively (Toki et al., 2019). Cytometry time-of-flight imaging mass cytometry (CyTOF) is another tool that has been used in conjunction with multiplexing to show that proximity of antigen-experienced cytotoxic T cells (CD8^+^CD45RO + Ki67+) to melanoma cells was associated with positive response to ICI (Moldoveanu et al., 2022). In the metastatic setting, multiplexed mass cytometry-based imaging has shown that enrichment of B cell patches and follicles with naïve-like TCF7+ T cells is a favorable predictive indicator of ICI response (Hoch et al., 2022).

## Artificial intelligence and multi-parameter biomarkers

While AI, a set of sophisticated algorithms and highly advanced machine learning tools to simulate some aspects of human intelligence, has greatly expanded its reach across all of medicine, it has demonstrated new potential horizons for melanoma biomarker development.

Machine learning, a subset of AI that involves computers improving performance from learned experience and pattern recognition, has been leveraged as an important tool for the identification of prognostic biomarkers. A study in 2022 utilized a machine learning classifier that accounted for multiple variables of TILs including cell type (e.g., tumor cells, immune cells) and area of interest (e.g., tumor, adjacent stroma) to validate the prognostic value of TILs for potential pathologist-independent use in future clinical trials. In this study, machine learning found that automated TIL score is prognostic in clinically-localized primary melanoma and may assist in isolating a subgroup of stage II patients with high recurrence risk. This will ultimately enable identification of patients who would likely benefit from adjuvant therapy (Aung et al., 2022). In another study, machine learning contributed to the development of immune diagnostic models to accurately classify melanoma patients from normal patients (Kulkarni et al., 2020; Du et al., 2022). Moreover, these authors could develop prognostic models to estimate composite risk score with clinical parameters to predict survival of over three to 5 years in melanoma patients. Patients can then be stratified based on these models into high versus low risk subgroups with different life expectancies (Du et al., 2022). A subsequent study, using machine learning, confirmed the prognostic value of TNM staging and also found that clinicopathological variables such as sex, tumor site, histotype, growth phase, and age, were linked to OS. The authors transformed their results into an online tool for prognostication for patients with melanoma (Cozzolino et al., 2023). Other AI techniques have been leveraged for biomarker discovery implicated in melanoma metastatic progression and have identified novel prognostic biomarkers (Miñoza et al., 2022).

Aside from prognostication, AI may process large amounts of available clinical and histopathologic data to aid physicians in determining the most favorable therapeutic choices for each patient and avoid treatments that are more likely to fail or lead to adverse events (Johnson et al., 2021; Guerrisi et al., 2022). A study has found that deep learning, another AI tool that uses algorithms modeled to operate similar to the human brain (i.e., artificial neural networks), applied to histology specimens and clinical data may predict ICI response in advanced melanoma (Johannet et al., 2021). Recent studies have integrated clinical outcomes and transcriptomic data from melanoma patients on ICI and have generated predictions for ICI treatment responses (Ahmed et al., 2022b; Kong et al., 2022). One study in particular was able to develop four machine learning models utilizing random-forest classification (RFC) incorporating clinical and genomic features (RFC7), differentially expressed genes (DEGs, RFC-Seq), survival-related DEGs (RFC-Surv) and a combination model. All models achieved high area under the curve (AUC), suggesting strong performances. These authors found that TMB, as well as the novel genes *GSTA3* and *VNN2*, were important features in predicting ICI response (Ahmed et al., 2022b). Studies have also found that, in addition to clinical and transcriptomic data integration, simple segmentation of melanoma whole slide pathology images using machine learning can indicate ICI predictive biomarkers (Johannet et al., 2021; Li et al., 2021; Grossarth et al., 2023). Segmentation analyzes images at the pixel level to classify specific melanoma cells on the slide and ignore uninvolved tissue. This AI-based method has even achieved high sensitivity in detecting morphological changes in *BRAF*-mutated melanomas, providing additional information on targeted therapies (Kim et al., 2022).

## Discussion

Melanoma is an aggressive skin cancer with rising yearly incidence. The growing field of biomarker detection in melanoma is very promising for determining prognosis and predicting treatment response. These biomarkers have tremendous implications for future therapeutic decision-making and drug development.

Currently, standard clinical care algorithms utilize TNM staging for prognosis. IHC has been able to elucidate many prognostic and predictive biomarkers including MART1/Ki-67, preferentially expressed antigen of melanoma (PRAME), makers of lymphovascular invasion (e.g., CD31/SOX-10) and mismatch repair (MMR) proteins, among many others (Torres-Cabala et al., 2020). However, a number of these markers are not routinely used in the clinic due to a variety of reasons, including lack of validation or accurate predictive potential (Diamandis, 2012). Thus, newer technologies are necessary for more robust analyses of biomarkers. For example, scRNA-seq and spatial transcriptomics have accounted for heterogeneity in melanoma which was a limitation of gene expression profiling of bulk tumor tissue. The latter technique has identified key genetic signatures such as *BRAFV600E* which have been considered when treating patients with ICI. However, it is possible that analysis of crosstalk between individual cells or the spatial influence of 1 cell on another may lead to identification of novel targets for treatment. Genomic immune-based (e.g., interferon) signatures have also stratified melanoma patients into low and high risk groups based on level of immune surveillance, which can further guide precision ICI. Subsetting these signatures based on single cell data may allow for improved accuracy.

Additional technologies that have elucidated cellular interactions in the TME include the use of multiplexed, quantitative IHC which has allowed for the analysis of multiple cellular phenotypes at a time, in addition to assessing proximity of individual cells to each other. In particular, multiplexing has better characterized TILs and their role in the TME.

AI-based analyses have also expanded biomarker discovery in melanoma. By simulating some aspects of human intelligence in a sophisticated and automated platform, these tools have the propensity to decrease inter-observer variability and error in order to more reliably quantify biomarker presence in patient samples based on integrated clinical and histopathologic data along with image analysis.

In the prognostic setting, the GEP test that classifies melanoma patients as Class 1 (low-risk) or Class 2 (high risk) for recurrence or metastasis is commercially available, which may allow clinicians to modify screening intervals and treatment regimens depending on a patient’s individual disease risk. However, studies have assessed the performance of this tool and while this GEP test generally identifies recurrence in patients with stage II disease, correctly identifying recurrence in stage I patients is poor, limiting its clinical utility (March et al., 2020). Thus, some of the aforementioned prognostic biomarkers will require further investigation for integration into standard AJCC staging and use in the clinic.

In the predictive setting, therapeutically targeting PD-1 and CTLA-4 correlate with clinical benefit. However, given intratumoral heterogeneity and limited ICI options, these markers are insufficient to capture the nature of all patient tumors. Additional biomarkers that have been well-explored such as the TMB and inflammatory mediators may soon be utilized in clinical settings. Newly discovered biomarkers such as antigen experienced cytotoxic T cells are likely to require additional evaluation, although preliminary data shows promise for predicting ICI treatment response.

While biomarkers may serve as independent prognostic or predictive indicators, a single biomarker is usually inadequate to precisely stratify patients. Thus, multimodal investigation of biomarkers using a combination of the techniques described while also prioritizing sensitivity, specificity and cost will be important for timely assessment of future patient risk and response.
